# Anti-Obesity Effect of the CB2 Receptor Agonist JWH-015 in Diet-Induced Obese Mice

**DOI:** 10.1371/journal.pone.0140592

**Published:** 2015-11-20

**Authors:** A. N. A. Verty, A. Stefanidis, A. J. McAinch, D. H. Hryciw, Brian Oldfield

**Affiliations:** 1 Department of Physiology, Monash University, Clayton, VIC 3800, Australia; 2 Centre for Chronic Disease Prevention and Management, College of Health and Biomedicine, Victoria University, St. Albans, VIC 3021, Australia; 3 Department of Physiology, The University of Melbourne, Parkville, VIC 3010, Australia; Universidad Miguel Hernández de Elche, SPAIN

## Abstract

The cannabinoid receptor 2 (CB2) is well known for its immune modulatory role. However, recent localisation of CB2 receptors in metabolically active tissue suggests that the CB2 receptor plays a significant role in energy homeostasis. This study was designed to investigate the impact of chronic CB2 receptor stimulation on food intake, body weight and mood. Lean male C57BL/6 mice were injected i.p. with the selective CB2 receptor agonist, JWH-015 (0.0, 1.0, 5.0 and 10.0 mg kg^-1^) to establish dose response parameters. Mice made obese following exposure to a diet consisting of 19.4 MJ/kg (4641 Kcal/kg) of energy (19.0% protein, 21.0% total fat, 4.7% crude fiber, and 4.7% AD fiber were given either vehicle or 10 mg/kg JWH-015. Impact on mood, food intake, body weight, plasma metabolites, expression of key metabolic proteins in the brown adipose tissue (BAT) and white adipose tissue (WAT), and markers of inflammation were measured. High dose (10 mg/kg) JWH-015 reduced food intake after 1, 2, 4, and 24 h in lean mice. When given to diet induced obese (DIO) mice, a 10 mg/kg dose of JWH-015 significantly reduced body weight compared to vehicle. This dose led to a shift in markers of lipid metabolism and inflammation in WAT consistent with lipolysis and improved immune response. Furthermore, JWH-015 (10 mg/kg) produced a transient reduction in food intake and significant reduction in fat mass and adipocyte cell size. Importantly, JWH-015 produced an anxiolytic response in the elevated plus maze while having no effect on immobility time in the forced swim test. It should be noted that though the 10 mg/kg dose produced positive effects on the obese state, the possibility that these effects are mediated via non-CB2 receptor mechanisms cannot be ruled out. These results demonstrate a role for CB2 receptors in modulating energy homeostasis and obesity associated metabolic pathologies in the absence of any adverse impact on mood.

## Introduction

The endogenous cannabinoid system has long been known as a potent modulator of appetite.[1] Most of this work has focused on the role of the cannabinoid receptor 1 (CB1) in food intake and energy homeostasis. Specifically, administration of cannabinoid-like compounds either intraperitoneally (i.p.) [2, 3] or into discrete hypothalamic nuclei [4] stimulate appetite. Furthermore, in food deprived animals the endogenous cannabinoid 2-arachidonylglycerol (2-AG) [5] is elevated in the hypothalamus and decreases following re-feeding, with normalization upon satiation [6]. Conversely, compounds that reduce endogenous CB1 receptor activity such as rimonabant [7] reduce appetite [8] and body weight in lean [9, 10] and diet-induced obese (DIO) [11, 12] rodents via a mechanism that involves increased energy expenditure [12]. Given the positive role of rimonabant on obesity, this drug was considered a promising anti-obesity pharmacotherapy. However, this promise was short lived and rimonabant was withdrawn from the market due its adverse psychiatric impact related to increased anxiety/depression and suicidal ideation [13, 14].

With the high profile demise of rimonabant, more attention has been paid to the other major category of cannabinoid receptor system, namely the CB2 receptors and its potential role in mediating energy homeostasis. This receptor system is believed to be primarily localized on immune cells and is well known for its role in mediating immune function (for a review see [15]). The recent localization of the CB2 receptor in brain regions mediating appetite [16, 17] and in peripheral metabolically active sites including the liver [18], adipose tissue [19–21], skeletal muscle [22], and pancreatic islet cells expressing insulin [23, 24] highlights the possible role of this receptor in energy homeostasis. The inhibition of CB2 receptor signaling either via the intracerebroventricular infusion or i.p. injection of the antagonist, AM630 produces a significant increase in food intake in non-obese rodents [16, 25, 26]. Similarly, the deletion of CB2 receptors leads to an age related obese phenotype characterized by increased food intake, body weight, and adipose tissue hypertrophy [27]. With regards to glucose homeostasis, acute administration of a CB2 receptor agonist JWH-133 improves glucose tolerance in non-obese rats given a glucose load [24] suggesting a role for this receptor in improving obesity associated diabetes. Importantly, the absence of CB2 receptors in brain regions affecting mood leaves open the possibility that this cannabinoid receptor may play a significant role in alleviating the obese condition while producing minimal psychological side effects [16, 21]. Taken together, these studies suggest a role for CB2 receptor stimulation in attenuating food intake and body weight gain without an adverse impact on mood.

Despite these promising observations, the effect of chronic CB2 receptor stimulation on obesity and its associated pathologies is poorly characterized. Therefore, this study was designed to further assess whether chronic administration of a CB2 receptor agonist could attenuate body weight gain using a rodent model of DIO. In addition to body weight gain, impact on food intake, plasma metabolites, expression of key metabolic genes in brown adipose tissue (BAT) and white adipose tissue (WAT), and key immune related markers in the WAT were also measured. Importantly, this study examined whether long-term stimulation of CB2 receptors affects anxiety and depressive-like behaviors, characteristics that limited the applicability of CB1 receptor antagonists.

## Methods and Materials

### Animals and Drugs

Experimentally naïve male C57BL/6 mice were housed in opaque polypropylene cages with stainless steel wire lids. Cages were lined with dust-free wood chips and were housed in a climate-controlled (20–23°C) room maintained on a 12-h light, 12-h dark cycle (lights off at 1200 hours). All animals were treated in accordance with the “Principles of Laboratory Animal Care” (NIH publication No. 85–23, revised 1985) and the Australian Code of Practice for the Care and Use of Animals for Scientific Purposes. This study was reviewed and approved by the Monash University School of Biomedical Science animal ethics committee.

JWH-015 [(2-Methyl-1-propyl-1H-indol-3-yl)-1-naphthalenylmethanone, Tocris Biosciences, Bristol, UK] and AM630 [6-lodo-2-methyl-1-[2-(4-morpholinyl)ethyl]– *1H –*indol-3-yl](4-methoxyphenyl)methanone, Tocris Biosciences, Bristol, UK] were dissolved in a solution containing 10% DMSO (Dimethyl sulfoxide; Sigma-Aldrich, Sydney, New South Wales, Australia) in distilled water. Vehicle consisted of 10% DMSO in distilled water. Animals were injected i.p. with either the vehicle or JWH-015 and vehicle or AM630 at the start of the dark phase. JWH-015 and AM630 binds more readily to the CB2 than to the CB1 receptor (28x and 165x, respectively, greater affinity for CB2 over CB1 receptors) and functions as a selective CB2 receptor agonist in functional assays [28, 29].

### Dose dependent effect of JWH-015 on food intake

This experiment was conducted in order to determine the dose of JWH-015 most effective in suppressing food intake. Specifically, 8 week-old male C57BL/6J mice (Animal Resource Centre, Perth; N = 10) were individually housed and maintained on laboratory chow diet (GR2 rat and mouse cubes; Ridley AgriProducts, Pakenham, Victoria, Australia). Bottles filled with tap water were available at all times. Two days before the start of drug treatment all mice were habituated to the experimental protocol by injecting them with vehicle (10% DMSO in distilled water) 30 min prior to the start of the dark cycle. During this time no food was available to the animals. At the beginning of the dark cycle (i.e., 30 min post injection) ≈20g of food was presented. Food consumed was measured at 1, 2, 4, and 24 h post injection. On day 3, prior to the start of drug treatment, mice were food deprived for 12 h in order to obtain a higher baseline of food intake in vehicle treated animals. Next, mice were injected 30 min prior to the start of the dark cycle with either vehicle or JWH-015 (1.0, 5.0, or 10.0 mg/kg). At the start of the dark cycle, that is 30 min following vehicle or drug treatment a pre-weighed (≈ 20g) amount of food was presented in their home cages. The total amount of food consumed was determined at 1, 2, 4, and 24 h post injection. The measurement of food intake during the dark cycle for habituation of drug treatment sessions was conducted under red light. The doses of JWH-015 were selected based on a previous study using the same drug [30]. Drug or vehicle was injected every 48 h in order to allow sufficient washout time between doses. On the day between drug sessions, animals were injected with vehicle and treated as per the experimental protocol and total food consumed measured. Food intake was measured during this time in order to ascertain drug wash out.

### Effect of combined AM630 and JWH-015 on food intake

In order to establish that JWH-015 suppresses food intake via the CB2 receptor, we co-administered JWH-015 with the CB2 receptor antagonist AM630 to 8 week-old male C57BL/6J mice (Animal Resource Centre, Perth; N = 10). The experimental protocol was similar to that described above with the exception that AM630 (5 mg/kg) was injected 15 min prior to JWH-015 (10 mg/kg). Food intake was measured 1, 2, 4, and 24 h post injection.

### Effect of JWH-015 on food intake, body weight, and energy efficiency in DIO mice

Once the effective dose of JWH-015 was determined, 6 week-old male C57BL/6J mice (Animal Resource Centre, Perth; N = 12) were maintained on a high fat diet (HFD) (High Fat Rodent Diet, ID SF00-219, Specialty Feed, Glen Forrest, WA, Australia) for 20 weeks to induce DIO. This diet provided 19.4 MJ/kg (4641 Kcal/kg) of energy (19.0% protein, 21.0% total fat, 4.7% crude fiber, and 4.7% AD fiber). During the 20-week period of maintenance on the HFD, body weight was measured weekly. DIO animals were divided into two equal groups receiving vehicle or 10.0 mg/kg JWH-015 injected daily for a period of 21 days at the start of the dark cycle. During this time, 24 h food intake and body weight were measured daily. Feed efficiency was calculated over 21 days as body weight lost per kilocalorie ingested in mice treated with vehicle or JWH-015.

### Blood chemistry

At the end of the 21-day treatment period, animals were food deprived for 6 h and deeply anaesthetised with isoflurane and blood samples, taken via cardiac puncture, were collected in 1 ml EDTA tubes (Greiner Bio-One GmbH, Frickenhausen, Germany). Blood samples were then centrifuged at 4000g for 5 min at 4°C. Plasma total non-esterified fatty acids (NEFA; Wako Diagnostics, Richmond, VA, USA), triglycerides (TG) (Roche Diagnostics, Indianapolis, IN, USA), insulin (Crystal Chem Inc, Downers Grove, IL, USA), and aspartate transaminase (AST, BioAssay Systems, Hayward, CA, USA) were measured according to the manufacturers protocol.

In addition, the impact of acute treatment with vehicle and JWH-015 on glucose metabolism was measured via a glucose tolerance test (GTT). JWH-015 and vehicle were injected at a volume of 5 ml/kg^1^. Drug and vehicle were given 1 h before commencement of glucose tolerance tests. Each mouse received 1 g/kg glucose in a 25% w/v solution in saline in a volume of 5 ml/kg^-1^. Blood glucose was measured using a handheld glucometer (Accu-chek, Roche) before and at 15, 30, 45, 60, 75, and 90 min post glucose administration.

### Markers of thermogenesis, lipid metabolism, inflammation, and hypothalamic feeding peptides

Following blood collection, animals were decapitated and brown adipose tissue (BAT), inguinal white adipose tissue (iWAT), and retroperitoneal white adipose tissue (rWAT) were removed. The white fat pads represented subcutaneous and abdominal depots respectively. The BAT, rWAT, and iWAT were weighed and snap frozen and stored at -80°C for further analysis.

Protein from the BAT and rWAT was extracted as previously described [31]. Next, 10 μg protein (BAT) and 15 μg protein (rWAT) was loaded onto a Bio-Rad Mini-PROTEAN TGX Precast Gel (Bio-Rad Laboratories, Hercules, CA) and transferred onto an Immuno-Blot nitrocellulose membrane (Bio-Rad Laboratories, Hercules, CA). The membrane was blocked with 5% BSA in Tris-buffered saline/1% Tween 20 (Sigma, St Louis, USA) for 1 h and incubated overnight at 4°C, in the case of protein extracted from BAT, in a primary antibody raised against uncoupling protein 1 (UCP1, Santa Cruz Biotechnology, Santa Cruz, CA, diluted 1:1000). Extracts from WAT were incubated in antisera raised against Adipose Tissue Triglyceride Lipase (ATGL; Cell Signaling Technologies, Danvers, MA, USA, Rabbit polyclonal; diluted 1:1000), interleukin 10 (IL-10) (Santa Cruz Biotechnology Inc, Santa Cruz, CA, USA, Goat polyclonal; diluted 1:500), tumor necrosis factor-alpha (TNF-α) (Santa Cruz Biotechnology Inc, Santa Cruz, CA, USA, Goat polyclonal; diluted 1:500), and protein kinase A (PKA) RIIβ (BD Transduction Laboratories, Lexington, U.K., Mouse monoclonal; diluted 1:3000). Following secondary antibody incubation using anti-rabbit HRP (1:4000 for ATGL), anti-goat HRP (1:4000 for UCP1, IL-10, and TNF-α), and anti-mouse HRP (1:4000 for PKA RIIβ), protein expression signals were visualized by chemiluminescence using the Pierce ECL Western Blotting Substrate (Thermo Scientific, Rockford, IL, USA) on the Bio-Rad ChemiDoc system (Bio-Rad Laboratories, Hercules, CA). Relative densities of protein bands were assessed using Image J software (National Institutes of Health, Bethesda, MD).

### Adipocyte area

Following dissection of rWAT and iWAT, a small piece of each of the fat depots was immersed in Bouin’s solution overnight and then transferred to 70% ethanol and stored at 4°C. Tissue was fixed and embedded with a random orientation in paraffin and 10-μm sections were stained with haematoxylin and counterstained with eosin. For each sample, the size of the adipocytes was determined in 80 ± 6 adipocytes by measuring the diameter of the cell obtained from four sections of tissues taken in vehicle and JWH-015 treated animals. Cell diameter was determined at x200 magnification using a Nikon Eclipse *Ti* (Coherent Scientific, Hilton, SA, Australia). Adipocytes with irregular cell size were excluded from the measurement.

### Effect on forced swim test for depression and elevated plus maze for anxiety-like behaviors

To determine the impact of JWH-015 on mood, another cohort of 5-month-old mice (N = 10/group) were maintained on standard laboratory chow and injected with vehicle or JWH-015 (10.0 mg/kg/day) for a period of 21 days. Five month old mice were used in order to ensure that these animals were age matched to the DIO mice at the start of drug treatment. Following the 21-day treatment, mice were first tested for 7 min in the elevated plus maze test for activity and anxiety in a novel environment. The plus maze was made of wood, painted black and was raised 50 cm above the floor. It consisted of two opposite open arms of 50 cm x 15 cm and two closed arms of the same dimensions with 15 cm high walls. Each mouse was placed in the centre of the plus maze and filmed and later scored for the number of entries into and percentage time spent in each of the open and closed arms and the incidences of vertical exploration (rearing). An experimenter blind to the treatment conducted the scoring. The animal was regarded as having moved into an arm when all four paws had crossed the threshold of the arm. The maze was thoroughly cleaned with 70% ethanol between trials.

The following day, the same animals as those tested in the elevated plus maze were tested in the forced swim test. The forced swim test was conducted in a cylindrical plastic drum, 45 cm in diameter, filled to a depth of 40 cm with warm tap water (approximately 30°C). Animals were videotaped and later scored by an experimenter blind to the treatment for time spent in each of the three behaviours: (1) climbing, defined as active forepaw contact with the wall; (2) swimming, defined as an active attempt to keep the head above water by moving the limbs, or forward propulsion under the water; and (3) floating, defined as immobile behaviour excepting the very minimal movements required to keep the head above water. After the trial the mice were carefully dried and returned to their home cages.

### Statistical analysis

Daily food intake (g) and body weight (g) were used as dependent variables and analysed separately. The data for total food intake and body weight was averaged for each day for each treatment group to give a daily variation in food intake and body weight. The food intake and body weight data was analysed using a repeated measures (treatment by day) ANOVA with day as the repeated factor. Where significant main effects were found, pairwise comparisons were conducted using Bonferroni adjustments for multiple comparisons. Mauchly’s W was computed to check for violations of the sphericity assumptions. When Mauchly’s W test was significant, the Greenhouse-Geisser correction was applied. For JWH-015 and AM630 dose response and combined JHW-015 and AM630 data was analysed using a one-way ANOVA. For western blot, iWAT, rWAT, and BAT weights, adipocyte cell size, plasma NEFA, triglycerides, insulin, AST levels, and GTT were analysed using a *t*-test to determine if the total food consumed, protein expression, fat pad weights, and NEFA, triglycerides, insulin, AST levels, and GTT of the various groups were significantly different compared to vehicle.

## Results

### Dose dependent effect of JWH-015 on food intake

**Fig 1:**
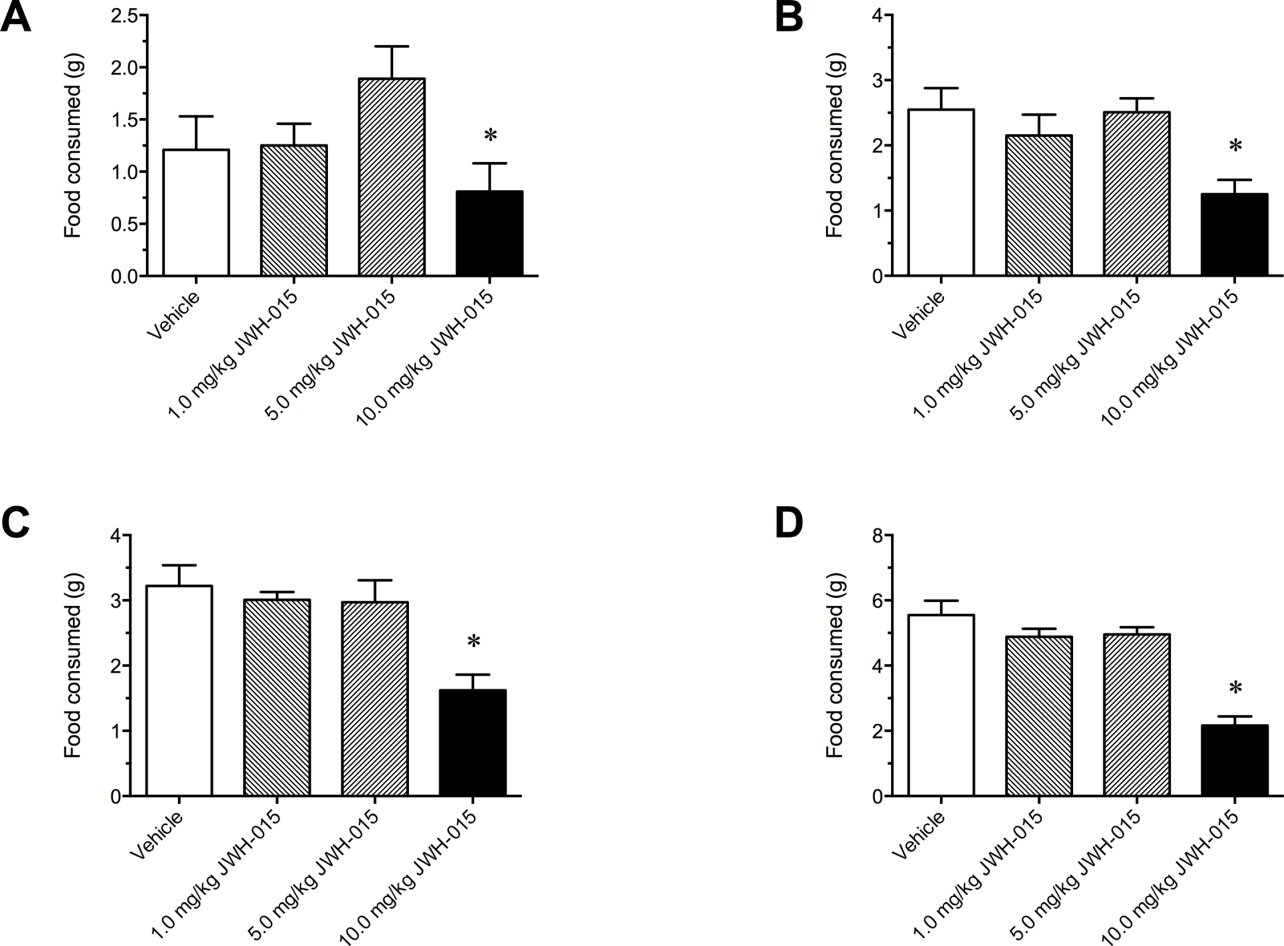
Laboratory chow consumed in 12 h food deprived mice at the 1 (A), 2 (B), 4 (C), and 24 (B and D) h measurement intervals after intraperitoneal administration of vehicle or 1.0, 5.0, or 10.0 mg/kg of JWH-015. Data represent means ± SEM. *P<0.05, significantly different from Vehicle, 1.0 and 5.0 mg/kg JWH-015.

The mean quantity of food consumed prior to JWH-015 treatment did not differ significantly compared to vehicle. As a result there was no significant difference in the mean body weights of the JWH-015 and vehicle treatment groups at the start of the experiment. In order to determine the anorectic dose of JWH-015, animals were administered either vehicle, 1.0, 5.0, or 10.0 mg/kg JWH-015 just before the start of the dark phase and food consumed at 1, 2, 4, and 24 h post injection was measured. The total food consumed at the 1, 2, 4, and the 24 h measurement intervals was not affected by the 1.0 or the 5.0 mg/kg dose of JWH-015 when compared to vehicle. Food intake was significantly depressed at the 10 mg/kg dose of JWH-015 compared to vehicle at every measurement interval (1 h [F (3, 36) = 25.30, P < 0.001], 2 h [F (3, 36) = 48.00, P < 0.001], 4 h [F (3, 36) = 73.80, P < 0.001], and 24 h [F (3, 36) = 39.20, P < 0.001]) (Fig 1).

### Effect of combined AM630 and JWH-015 on food intake

**Fig 2:**
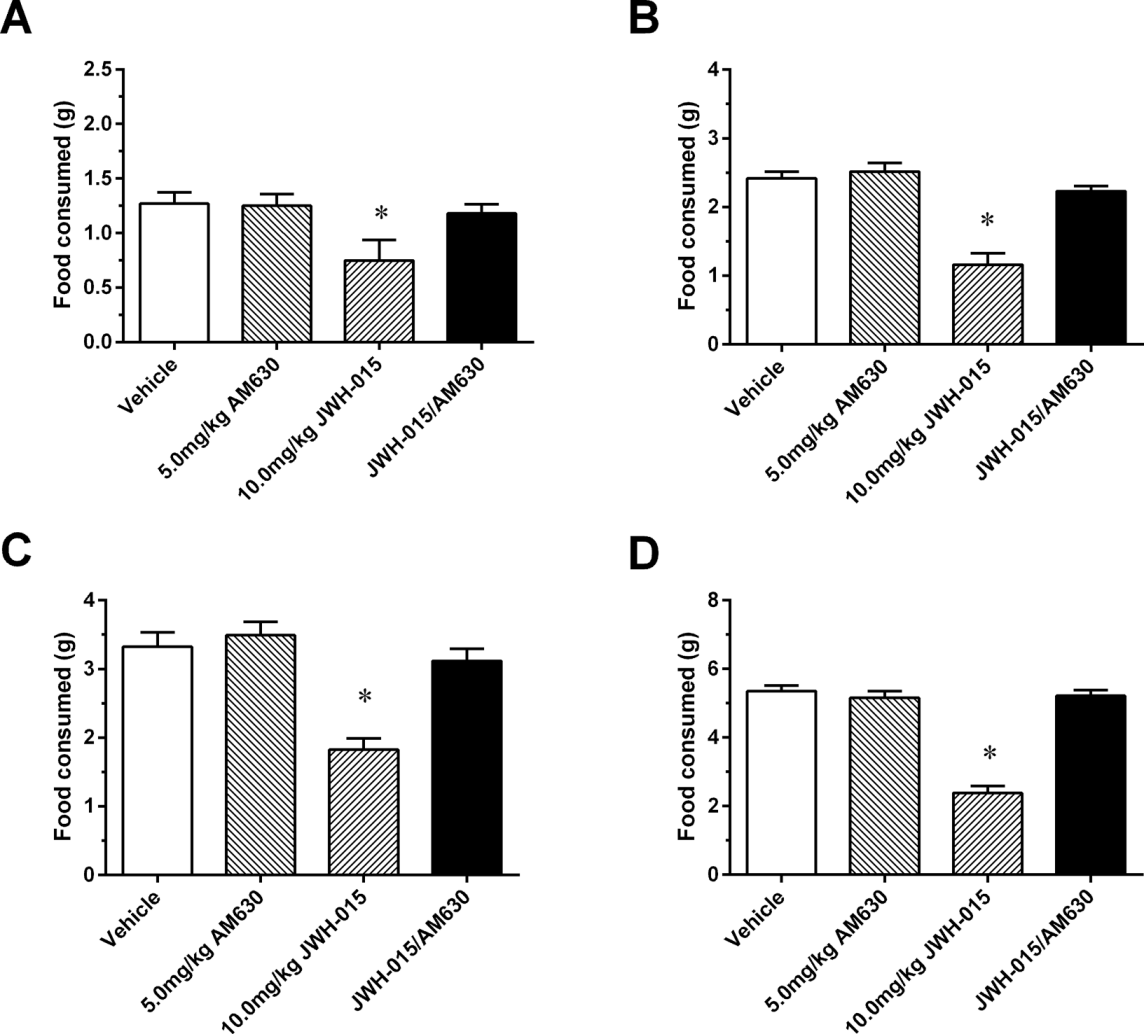
Laboratory chow consumed in 12 h food deprived mice at the 1 (A), 2 (B), 4 (C), and 24 (B and D) h measurement intervals after intraperitoneal administration of vehicle, 5mg/kg AM630, 10mg/kg JWH-015 or AM630 and JWH-015. Data represent means ± SEM. *P<0.05, significantly different from Vehicle.

In order to more definitely assess the involvement of CB2 receptors in the anorectic effects of JWH-015, we administered the CB2 receptor antagonist AM630 (5.0 mg/kg) 15 min prior to JWH-015 (10 mg/kg) treatment. The 5.0 mg/kg dose of AM630 was chosen as the dose response study with AM630 (vehicle, 1.0, 5.0, and 10.0 mg/kg) showed no effect on food intake. When co-administered with JWH-015 AM630 (5.0 mg/kg) showed a significant reversal of JWH-015 (10 mg/kg) induced suppression food intake at every measurement interval (1h [F (3,31) = 24.52, P < 0.001], 2h [F (3,31) = 159.6, P < 0.001], 4h [F (3,31) = 113.9, P < 0.001], 24h [F (3,31) = 335.7, P < 0.001]. (Fig 2)

### Effect of JWH-015 on food intake, body weight, and energy efficiency in DIO mice

**Fig 3:**
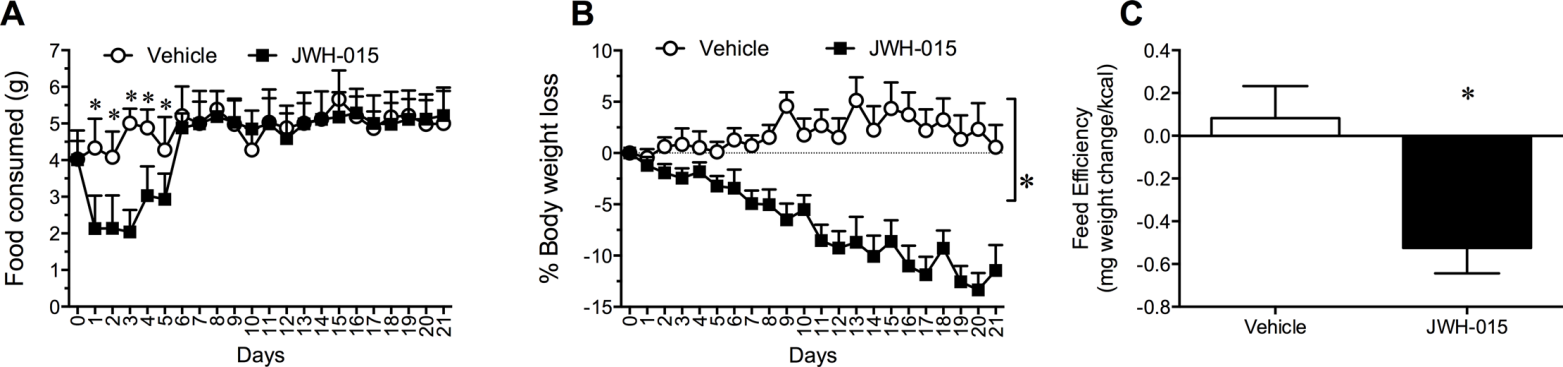
24 h food intake (A), percentage weight loss (B), and energy efficiency (C) in diet induce obese mice following daily intraperitoneal administration of Vehicle or 10 mg/kg/day of JWH-015 for 21 days. Data are means of total food intake (A) and percentage weight loss (B) of all animals in each group for each day. Error bars represent the SEM. *P<0.05, significantly different from Vehicle.

Following determination of the effective dose of JWH-015 (10.0 mg/kg), this dose was then administered for 21 days to DIO mice and its impact on food intake and body weight was determined. Chronic treatment with JWH-015 produced a profound reduction in 24 h food intake when compared to vehicle [F (3, 20) = 22.64, P < 0.001] (Fig 3A). Interestingly, food intake was significantly depressed for the first 6 days out of the 21-day treatment period [F (36, 240) = 6.62, P < 0.001] (Fig 3A) while body weight continued to be depressed compared to vehicle for the entire duration of JWH-015 treatment [F (1, 15) = 23.66, P < 0.001] (Fig 3B). It is noteworthy that following administration of JWH-015, body weight loss did not appear to plateau when treatment was terminated on day 21 (Treatment by time interaction [F (15, 128) = 21.68, P < 0.001]) (Fig 3B). Feed efficiency (weight gain per kilocalorie ingested) was calculated for the entire 21-day treatment period. The feed efficiency ratio, indicative of the animals’ efficiency in converting feed mass into body mass, was significantly reduced by JWH-015 [*t*_8_ = 8.32, P < 0.001] when compared to vehicle indicative of increased energy expenditure (Fig 3C).

### Changes in plasma NEFA, TG, insulin, AST, and GTT

**Fig 4:**
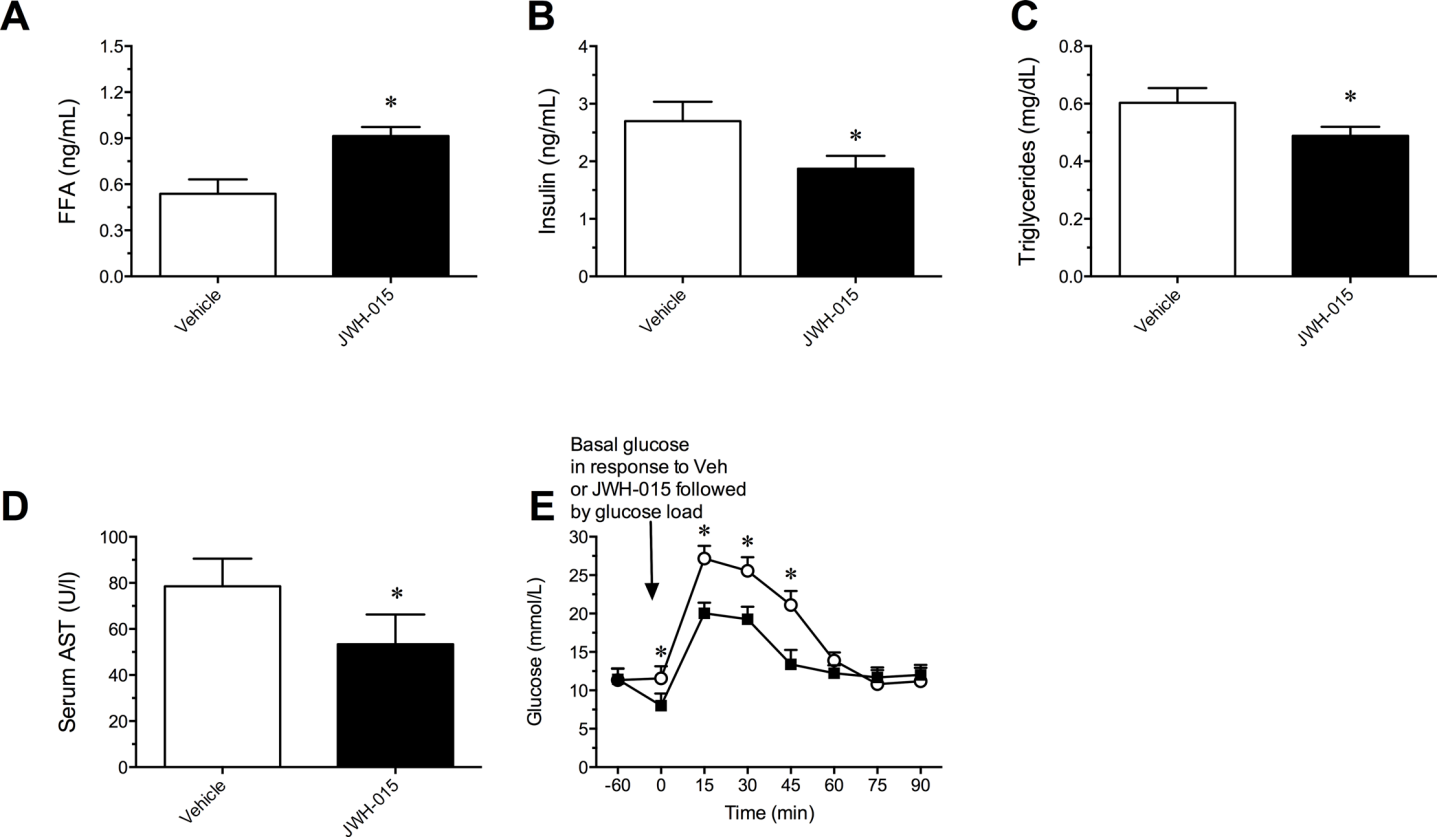
Change in plasma levels of non-esterified fatty acid (NEFA) (A), insulin (B), triglycerides (C), Aspartate Aminotransferase (AST) (D), and glucose tolerance test (GTT) (E) following daily intraperitoneal administration of Vehicle or 10 mg/kg/day JWH-015 for 21 days. JWH-015 was administered 60 min prior to the injection of a glucose load. Data represent means ± SEM. *P<0.05, significantly different from Vehicle.

The JWH-015 induced reduction in body weight lead to a significant reduction in plasma levels of FFA [*t*_10_ = 8.32, P < 0.001], TG [*t*_10_ = 4.69, P < 0.001], insulin [*t*_10_ = 5.00, P < 0.001], and AST [*t*_10_ = 6.19, P < 0.01] when compared to vehicle (Fig 4).

Acute administration of JWH-015 produced a significant improvement in glucose clearance in the GTT [F (7, 80) = 92.74, P < 0.001] (Fig 4E) compared to vehicle. The GTT reached significance at 15 (P < 0.001), 30 (P < 0.001), and 45 (P < 0.001) min measurements in mice.

### Adipose tissues mass and adipocyte cell size

**Fig 5:**
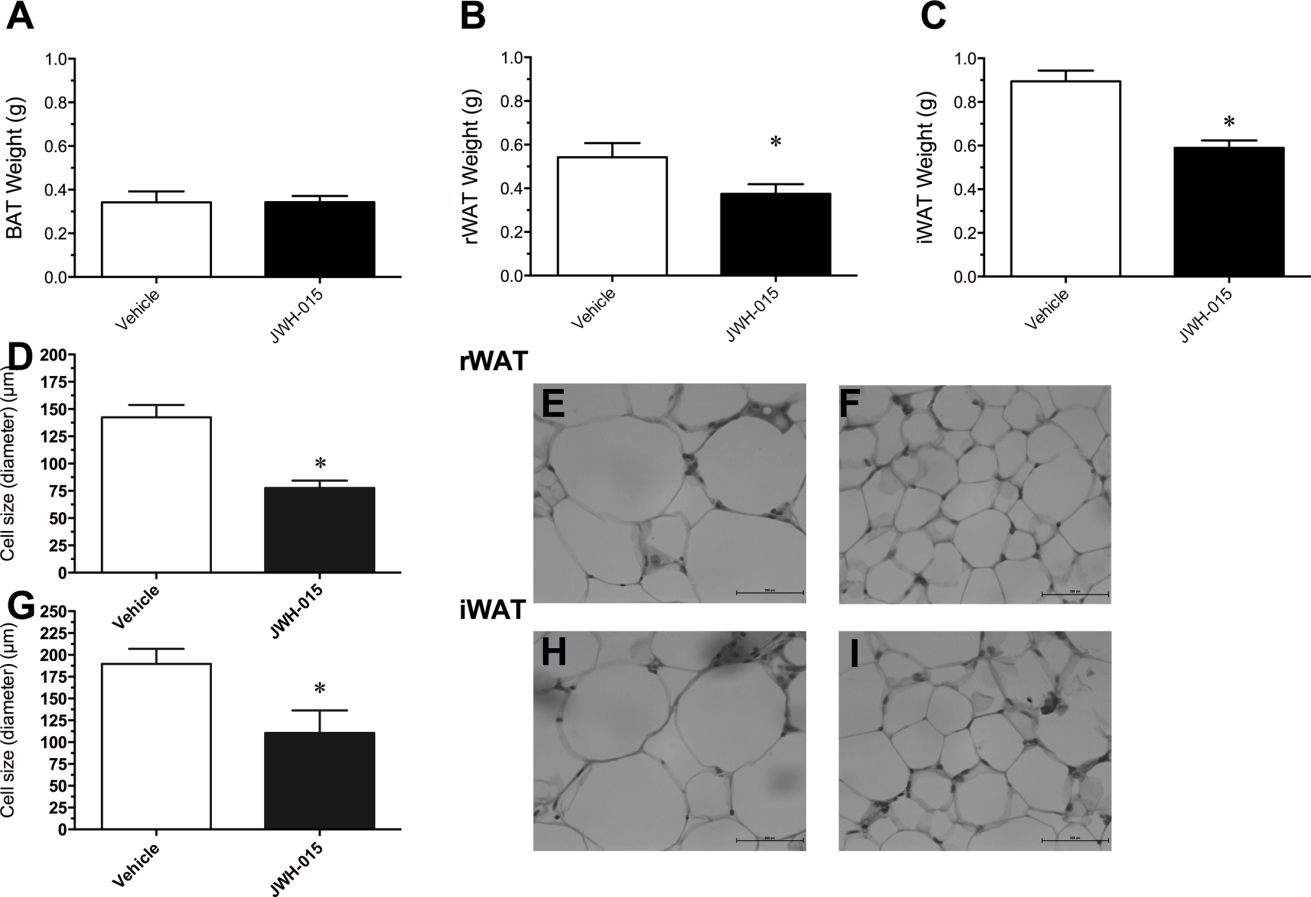
Change in brown adipose tissue (BAT) (A), retroperitoneal white adipose tissue *(*rWAT) (B), and inguinal white adipose tissue (iWAT) (C) weights, changes in adipocyte cell size in the rWAT (D) and iWAT (G), and distribution of adipocytes in abdominal (E-F) and subcutaneous (H-I) fat pads following daily intraperitoneal administration of Vehicle or 10 mg/kg/day JWH-015, respectively, for 21 days. Data for brown adipose tissue (BAT) (A), retroperitoneal white adipose tissue *(*rWAT) (B), inguinal white adipose tissue (iWAT) (C), and cell size in the abdominal and subcutaneous fat pads represent means ± SEM. *P<0.05, significantly different from Vehicle. Scale bar = 200μm.

The fat mass of the rWAT [*t*_10_ = 7.97, P < 0.001] (Fig 5B) and iWAT [*t*_10_ = 2.71, P < 0.001] (Fig 5C) was significantly lower in the animals chronically injected with JWH-015 compared to vehicle. Importantly, CB2 receptor stimulation via JWH-015 reduced fat mass by 40% (rWAT) and by 33% (iWAT) compared to vehicle. However, JWH-015 treatment did not significantly affect fat pad mass of the BAT (Fig 5A).

The reduced rWAT and iWAT weights were commensurate with changes with adipocyte cell size. Specifically, the mice receiving JWH-015 treatment showed a significantly reduced adipocyte cell size in the rWAT [*t*_10_ = 12.08, P < 0.001] (Fig 5D) and iWAT [*t*_10_ = 6.24, P < 0.001] (Fig 5G) when compared to vehicle. A representative image of the rWAT and iWAT is shown in (Fig 5E, 5F 5H & 5I).

### Markers of thermogenesis and lipid metabolism

**Fig 6:**
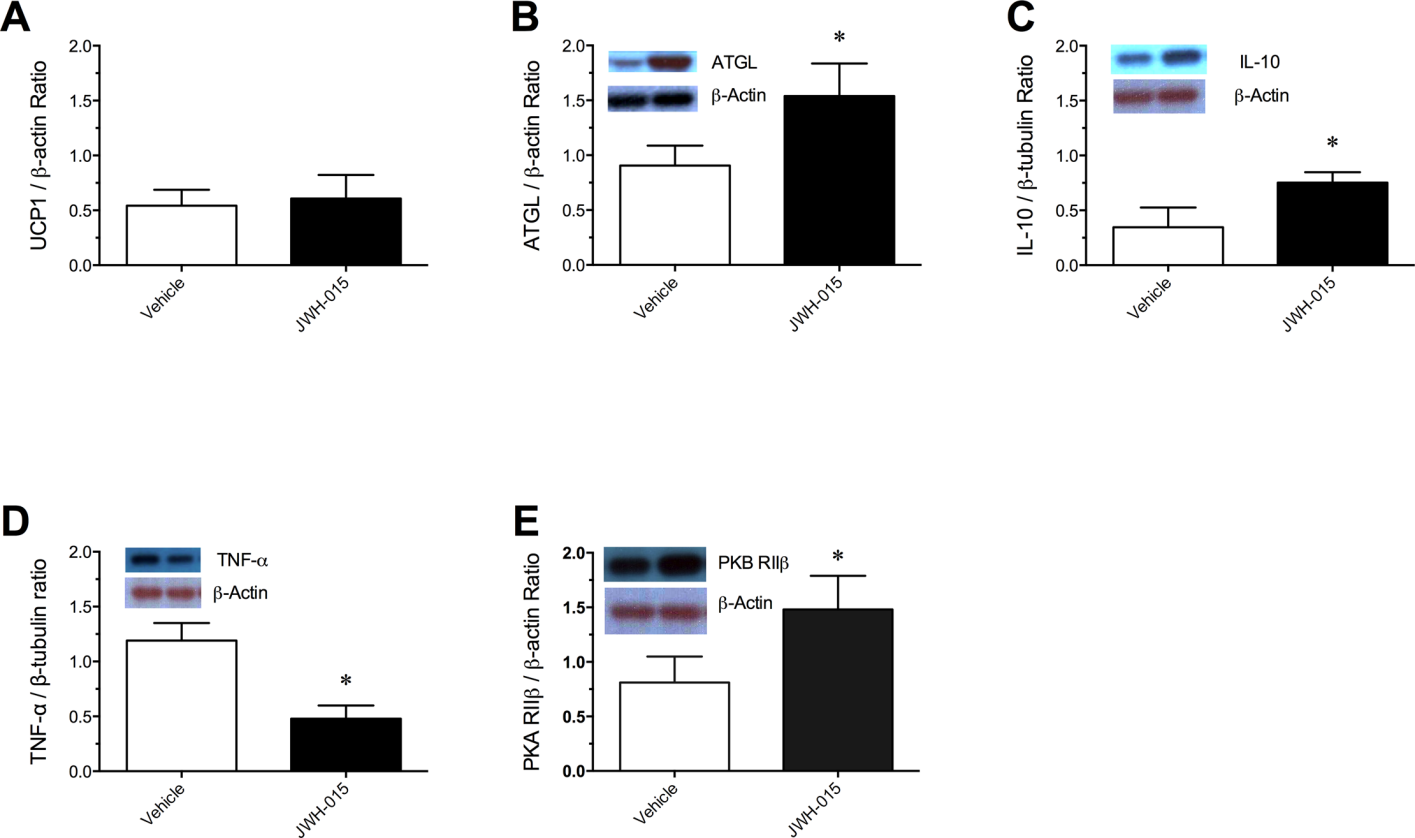
Western blot detection of Uncoupling protein 1 (UCP1) (A) protein expression in the brown adipose tissue, and adipose tissue triglyceride lipase (ATGL) (B) interleukin-10 (IL-10) (C), tumor necrosis factor-alpha (TNF-α) (D), and protein kinase A (PKA) RIIβ protein expression in the abdominal white adipose tissue from diet induced obese mice treated with Vehicle or 10 mg/kg/day JWH-015 for 21 days. Representative western blot from a single animal following the above treatments. Signals from each animal quantified and peptide expression shown as a ratio of housekeeping gene β-Actin (Uncoupling protein 1, adipose tissue triglyceride lipase, and protein kinase A RIIβ) and with β-tubulin (interleukin-10 and tumor necrosis factor-alpha). Results are expressed as mean ± SEM. *P < 0.05, significantly different from Vehicle.

Western blot analysis was used to detect changes in markers of thermogenic activity (UCP1 protein) in BAT and lipolysis (ATGL) in rWAT tissue of DIO mice treated with vehicle or JWH-015. A significant increase in ATGL [*t*_10_ = 4.46, P < 0.01] expression was seen in response to JWH-015 compared to vehicle (Fig 6). Given that obesity is associated with chronic low-grade inflammation, we examined the expression of pro- and anti- inflammatory cytokines TNF-α and IL-10 respectively. Results revealed that, compared to vehicle, JWH-015 treatment produced a significant increase in IL-10 [*t*_10_ = 4.88, P < 0.01] and a significant decrease in TNF-α expression [*t*_10_ = 8.70, P < 0.01] (Fig 6). In order to explore the mechanisms via which JWH-015 would affect lipolysis in the rWAT, we examined the expression of the cyclic AMP dependent protein kinase A (PKA) regulatory subunit RIIβ. Results revealed a significant increase of PKA RIIβ in mice treated with JWH-015 compared to vehicle group [*t*_10_ = 11.01, P < 0.01]. Interestingly, no impact of JWH-015 treatment was seen on the expression of UCP1 expression in the BAT.

### Effect of JWH-015 on behaviour observed in an elevated plus maze and Porsolt forced swim test

**Fig 7:**
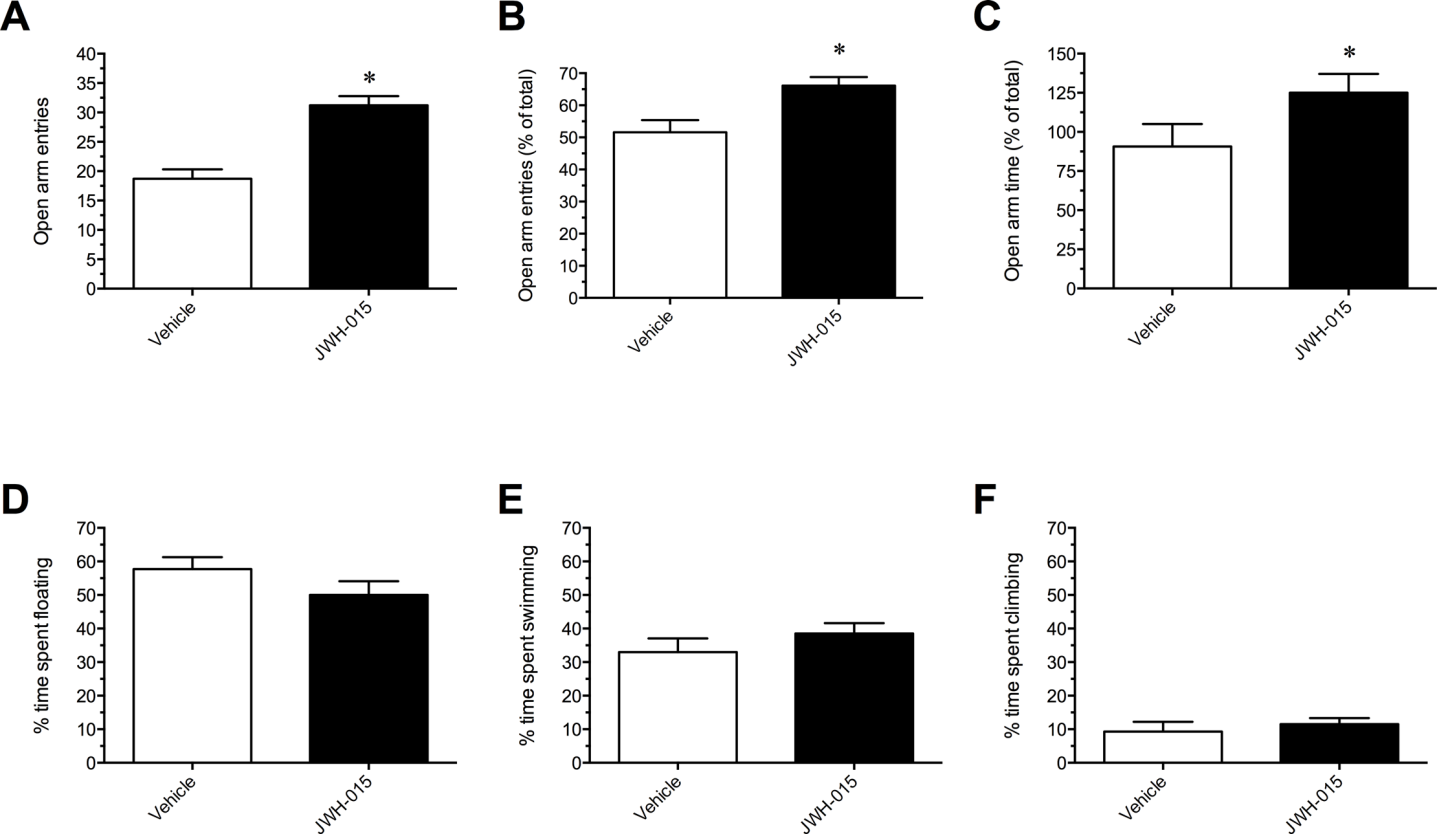
Forced swim test (A-C) and elevated plus maze (D-E) behavior in mice treated with Vehicle or 10 mg/kg/day JWH-015 for 21 days. Results are expressed as mean ± SEM. *P < 0.05 significantly different from Vehicle.

Behavior in the elevated plus maze was significantly affected by JWH-015 treatment. Mice treated with JWH-015 (10.0 mg/kg/day) entered the open arms significantly more often than vehicle treated mice [*t*_18_ = 17.47, P < 0.001] (Fig 7A). Significant differences between the vehicle and JWH-015 treated mice were revealed when open arm entries were expressed as a percentage of total entries to account for differences in locomotion [*t*_18_ = 9.84, P < 0.001] (Fig 7B). Similarly, time spent in the open arms expressed as a percentage of the total time spent in the open arms was also significantly different compared to vehicle treated mice [*t*_18_ = 5.84, P < 0.001] (Fig 7C). No effect of JWH-015 was observed in the “depressive like” behaviors in forced swim test when compared to vehicle treated animals.

## Discussion

The major findings of this study demonstrate for the first time that chronic administration of a CB2 receptor agonist produces a profound reduction in body weight gain in DIO mice despite only a transient reduction in food intake and no change in UCP1 expression in BAT. The effects of JWH-015 on body weight loss are associated with a significant increase in markers of lipolysis in the rWAT. The positive impact on weight loss in response to JWH-015 treatment induced a reduction in the fat mass of the rWAT and iWAT that is commensurate with a change in adipocyte cell size in these depots. This reduction in body weight was accompanied by increased levels of fasting induced plasma NEFA, and reduction in fasting induced insulin, TG, and AST. In addition, JWH-015 treatment to DIO mice resulted in an increased expression of the anti-inflammatory cytokine IL-10 and reduced expression of the pro-inflammatory marker TNF-α in the rWAT. Furthermore, JWH-015 administration improved glucose clearance rates following administration of a glucose load. Most importantly, the effect of JWH-015 on food intake and body weight was independent of any adverse outcomes on anxiety- and depressive-like behaviours using the elevated plus maze and Porsolt forced swim test, respectively.

The present findings with regards to a reduction in feeding following acute administration of a CB2 receptor agonist is in agreement with previous studies [26] and shows a reduction in food intake at the highest dose tested. This finding further supports the role of the CB2 receptor in mediating energy balance. The mechanisms via which CB2 receptor ligands mediate food intake and body weight are not yet clear. One possibility is that CB2 receptors act via the hypothalamic receptors to affect energy intake and expenditure. Indeed, JWH-015 produces a significant elevation of Fos immunoreactivity in the paraventricular, lateral, ventromedial, and arcuate nuclei of the hypothalamus [12]. In addition, CB2 receptors are localised within these regions [16, 32] and over expression of CB2 receptors suppress levels of orexigenic peptides neuropeptide Y, and galanin in the hypothalamus [32].

The data presented here show for the first time that the administration of JWH-015 produces a profound reduction in body weight after a transient reduction in food intake using a non-genetic model of obesity. These findings may suggest a role for energy expenditure as a possible mechanism underlying the anti-obesity effect of JWH-015; however, there is no discernible effect on BAT thermogenesis. This lack of any effect of UCP1 expression following chronic JWH-015 treatment is in agreement with a previous study showing no change in UCP1 expression in CB2^-/-^ mice fed a high fat diet despite a significant increase in overall body weight [27]. While we cannot rule out a contribution for more global metabolic shifts or physical activity it is clear that there are JWH-015—induced changes in markers of lipolysis (ATGL) that are consistent with an increase in catabolic processes in the WAT which will enhance weight loss [33, 34]. The impact of CB2 receptor agonist treatment on catabolic processes is consistent with the localisation of CB2 receptors on white adipocytes [35, 36] and suggests that one of the roles of CB2 receptors could be to stimulate lipolysis in the WAT.

In order to determine the mechanism by which chronic CB2 receptor stimulation impacts on catabolic processes in the white adipose tissue we examined the role of the protein kinase A (PKA) pathway. Our data show that changes in lipolysis could be mediated via CB2 receptor effects on PKA expression in the WAT. It is well established that increased PKA activity particularly the RIIβ subunit is associated with a lipolytic response in the adipose tissue [37] and is implicated in body weight regulation [38] in both genetically obese ob/ob [39] and DIO mice [40]. Though not specifically tested in WAT, chronic CB2 receptor stimulation have been shown to increase the expression of PKA in immune cells [41]. It is important to note that acute stimulation of CB2 receptors leads to a decrease in cyclic AMP production. However, sustained stimulation leads to an elevation of cyclic AMP and a corresponding increase in PKA expression [41]. Hence, our data showing an increase in PKA expression is consistent with this finding and further suggests that the chronic administration of JWH-015 could stimulate PKA activity that inhibits lipogenesis and increases lipolysis resulting in reduced body weight.

The localization of CB2 receptors on metabolically active tissue such as liver [18], adipose tissue {Matias, 2006 #87;Murdolo, 2007 #48;Spoto, 2006 #18;Roche, 2006 #19}, skeletal muscle [22], and pancreatic islets [19, 23, 24] raises the possibility of a role for this receptor in mediating obesity linked conditions such as hyperinsulinemia and hypertriglyceridemia [42]. In our study, chronic JWH-015 treatment reduced plasma insulin levels and triglyceride levels. Given that plasma insulin and triglyceride levels are negatively correlated with the degree of adiposity [43], the JWH-015 induced weight loss may contribute to improvement in these plasma parameters. This effect appears to be due to a direct impact of JWH-015 treatment in glucose clearance rates in the glucose tolerance tests. This finding is not surprising given the localization of CB2 receptors on insulin containing β-cells [23], decrease in insulin secretion as demonstrated by reduced amplitude of glucose-induced [Ca^2+^]_i_ oscillation following CB2 receptor activation [23], and improved glucose homeostasis in lean rats given the CB2 receptor agonist JWH-133 [24]. Furthermore, loss of CB2 receptors in the pancreas induces glucose intolerance suggesting a functionally relevant role for pancreatic CB2 receptors [32]. Taken together, these findings demonstrate a beneficial impact of CB2 receptor activation on glucose tolerance in obesity.

In our study, NEFA levels were significantly increased in DIO mice treated with JWH-015 compared to vehicle. An increase in plasma NEFA concentration has been shown following release from adipose tissue as a result of enhanced lipolysis [44, 45]. This is in keeping with the current data showing a significant increase in markers of lipolysis in the WAT after JWH-015 treatment. In addition to insulin, TG, and NEFA, chronic JWH-015 also significantly reduced AST blood levels in the current study. AST is a metabolic enzyme expressed primarily in the liver where its elevation is one of the hallmarks of non-alcoholic fatty liver disease (NAFLD) that occurs with increasing obesity [42]. The reduction in AST levels seen following JWH-015 indicates an improvement in liver function and a possible reduction in obesity induced NAFLD and is consistent with the localization of CB2 receptors in the liver [18]. Our data raises the possibility that CB2 receptor activation plays a role in protecting the liver from damage in obesity. Indeed, a similar protective role for CB2 receptor activation has been demonstrated in a mouse model of alcohol induced hepatic liver injury [46]. Furthermore, CB2 receptor activation reduces inflammation and the generation of reactive oxygen species in hepatic Kupffer cells in ischemia/reperfusion (I/R) model of injury [47]. However, it is noteworthy that a previous study has shown increase in fatty liver disease following CB2 receptor stimulation [48]. The reasons for these divergent findings are not clear, however it is possible that the dose of JWH-015 used in the current may activate non-CB2 mechanism.

It is well established that obesity induced low grade inflammation is associated with elevated levels of pro-inflammatory cytokines such as TNF-α [49, 50] and reduced levels of anti-inflammatory cytokines such as IL-10 [50, 51]. Our data has shown that chronic CB2 receptor stimulation leads to improvement in obesity associated inflammation. Previous studies have also shown suppression of pro-inflammatory markers and stimulation of anti-inflammatory markers following chronic CB2 receptor stimulation [52]. It should be noted that elevated TNF-α levels are also associated with insulin resistance, hypertriglyceridemia [53, 54], and hepatic steatosis [55]. Conversely, IL-10 production has beneficial effects on insulin sensitivity [56] and protects against liver disease [57]. Improvement in obesity associated metabolic pathologies following JWH-015 treatment could be synergistically mediated via CB2 receptor mediated weight loss and improved inflammatory response. Indeed, a recent study in obese post-menopausal women has shown that weight loss associated reduction in inflammation is associated with improved glucose metabolism [58]. However, this hypothesis needs further testing.

It is noteworthy that previous studies using CB2 receptor knockout mice fed a high fat diet have shown reduced inflammation, body weight gain, and improved insulin sensitivity [27, 48]. This finding seems contrary to the established literature with regard to the increase in the expression of pro-inflammatory markers following CB2 receptor antagonism (for a review see, [59]) or knockout [60]. The reason for these different findings is not yet clear; however, it is possible that CB2 receptor antagonism improves inflammation and insulin sensitivity via CB2 receptor independent pathways. Indeed, in a recent study a CB2 receptor agonist induced improvement in insulin sensitivity was not blocked by a CB2 receptor antagonist but in fact insulin release was stimulated by the CB2 receptor antagonist [61].

Finally, the present study demonstrated an improvement in the mood associated with long-term CB2 receptor agonist treatment. Here, we show that chronic administration of JWH-015 had no effect on immobility time in the Porsolt forced swim test. The lack of any impact on immobility time demonstrates that CB2 receptors do not appear to have a role in depressive-like behaviours. Similarly, chronic JWH-015 treatment produced an anxiolytic profile in the current study as shown by a significant increase in the open arm entries in the elevated plus maze. These effects appear consistent with a recent study showing an absence of CB2 receptors in the parts of the brain associated with mood [16]. Our findings also complement a recent study in which CB2 receptor over expressing mice showed reduced anxiety in the elevated plus maze while having no effect on depression-like behaviours [32, 62]. Our data does not preclude any aversive mood related effects following acute CB2 receptor stimulation. However, it is important to note that there were no observable medium to long term impact on mood following chronic CB2 receptor agonism.

It is possible that the reduction in food intake seen after acute injection of JWH-015 (Experiment 1) could result from altered behaviours such as depressed locomotor activity, reduced exploratory behaviour, and catalepsy. This seems unlikely as previous studies have shown no change in these parameters [26, 63] in rodents using doses of JWH-015 similar to those used in the current study. Furthermore, cannabinoid receptors have been shown to have an impact on emesis and malaise in vomiting and non-vomiting species respectively (for a review see [64]). Given this, it is possible that the suppression of food intake seen following the acute administration of JWH-015 (Experiment 1) could result from a malaise/sickness response. In non-vomiting species such as rats and mice, CB1 antagonists/inverse agonist such as rimonabant [65] and AM251 [66] have been shown to be noxious and induce illness/malaise. While in vomiting species such as the house musk shrew, CB1 receptor agonists have been shown to be potent anti-emetic agents [67–70] while the CB1 antagonists produce emesis [71]. CB2 receptors localization in the dorsal vagal complex suggests a possible role for these receptors in the regulation of emesis and malaise [17].

The reasons for anorectic effect of JWH-015 are unclear. The possibility cannot be excluded that JWH-015 suppresses appetite and weight gain by activating non-CB2 receptors. Indeed, a recent study has shown JWH-015 also has a ~4x affinity for the CB1 receptor and activates these receptors on hippocampal neurons using experimentally relevant concentrations [72]. The possibility remains that JWH-015 has a biphasic effect at CB1 receptor with the dose (10 mg/kg/day) used in the current study. Furthermore, metabolism of JWH-015 produces over 20 metabolites, none of which have been pharmacologically characterised [73]. The impact of these metabolites on food intake and body weight gain could contribute to the effect seen here. It should be noted that there are significant pharmacokinetic and pharmacodynamic differences between lean and DIO mice [74]. These differences have not been evaluated with regards to CB2 receptor signalling however it is possible that the dose of JWH-015 though effective in suppressing food intake in lean (Fig 1) and DIO mice (Fig 2) may not be the optimal dose for use in DIO mice.

In summary, the present results using DIO mice demonstrate that CB2 receptor agonists are efficacious in reducing body weight and obesity associated metabolic parameters while having no effect on mood related behaviours. The positivity of these data needs to be tempered by the observation of adverse effects of JWH-015 treatment in previous studies in animal models and in humans. These particularly relate to an increase in alcohol preference in a model of chronic mild stress and a positive correlation between Q63R polymorphism of the CB2 receptor and alcoholism in humans [75]. Furthermore, CB2 receptor stimulation has also been shown to increase the population of slow/sluggish progressive sperm cells [76]. These potential adverse effects need to be taken into account while considering CB2 receptor agonists as possible pharmacological therapies for the treatment of obesity and associated metabolic disorders.
